# *In Utero* and Postnatal Exposure to High Fat, High Sucrose Diet Suppressed Testis Apoptosis and Reduced Sperm Count

**DOI:** 10.1038/s41598-018-25950-3

**Published:** 2018-05-16

**Authors:** Jiude Mao, Kathleen A. Pennington, Omonseigho O. Talton, Laura C. Schulz, Miriam Sutovsky, Yan Lin, Peter Sutovsky

**Affiliations:** 1 Division of Animal Sciences, University of Missouri, Columbia, MO 65211 USA; 2 Department of Obstetrics, Gynecology and Women’s Health, University of Missouri, Columbia, MO 65211 USA; 3 Department of Obstetrics and Gynecology, Baylor College of Medicine, Houston, TX USA; 4 Key Laboratory for Animal Disease Resistance, the Ministry of Nutrition of Education of China, Institute of Animal Nutrition, Sichuan Agricultural University, Ya’an, 625001 China

## Abstract

Obesity affects male fertility and maternal diabetes affects the offspring sperm epigenome. However, the effects of *in utero* exposure to maternal glucose intolerance in combination with postnatal high fat, high sucrose (HFHS) diet consumption on offspring spermatogenesis is not clear. The present study was designed to test these effects. One week before and during pregnancy, dams were fed either control or HFHS diet to induce gestational glucose intolerance, and returned to standard diet during lactation. Male offspring from each maternal group were split into control and HFHS-fed groups for eight weeks prior to sacrifice at 11, 19 or 31 weeks of age, and reproductive tissues were harvested for analysis of testicular germ cell apoptosis and sperm output. Postnatal HFHS diet suppressed spermatogonia apoptosis in all age groups and maternal HFHS diet reduced testosterone levels at 11 weeks. At 31 weeks of age, the postnatal HFHS diet increased body weight, and reduced epididymis weight and sperm count. The combination of *in utero* and postnatal exposure impacted sperm counts most significantly. In summary, HFHS diet during pregnancy puts male offspring at greater risk of infertility, particularly when combined with postnatal high fat diet feeding.

## Introduction

One in ten American couples of reproductive age is involuntarily infertile. Male infertility accounts for half of these cases. Body mass index (BMI) in men, an indicator of body fat, has been inversely associated with semen quality^1,2^ and fertility^3–5^. For example, the percentage of spermatozoa with abnormal morphology was doubled in men with BMI ≥35 kg/m^2^ compared to those with BMI in the normal range^6^. Total sperm count in overweight or obese men was lower compared with men with normal BMI^7^. The number of spermatozoa with DNA damage was also significantly higher in obese men than in normal weight men^2^. Accordingly, couples whose BMI was ≥35 kg/m^2^ have a longer time to pregnancy in comparison to leaner couples (BMI ≤25 kg/m^2^)^8^.

Paternal obesity and diabetes also impact the reproductive health of offspring. For example, in rodents, male obesity alters blastocyst development, embryo metabolism^9,10^, placental gene expression and methylation status^11^, and reduces semen quality^12^ and fertility of male offspring^13^. Paternal prediabetes induced by streptozotocin treatment alters global sperm cytosine methylation profiles and offspring insulin sensitivity^14^. Similarly, maternal obesity, and maternal type I and II diabetes have been shown to negatively impact the metabolic health of adult offspring^15–17^, as well as the sperm epigenome of adult offspring^18,19^.

Collectively, these studies show that exposure to an adverse maternal metabolic environment can impact male offspring, and this may in turn affect the metabolic and reproductive health of their offspring. However, the effect of *in utero* exposure to maternal diabetes on fertility of male offspring has not been studied. We have developed an animal model of gestational glucose intolerance, in which C57BL/6J dams are fed a high fat, high sucrose diet from 1 week pre-gestation through the end of pregnancy^20^. Under this acute HFHS exposure, dams fail to expand beta cell numbers during pregnancy, leading to an inadequate insulin response to glucose challenge, and glucose intolerance during mid-late pregnancy^20^. Here, using this model, we ask whether maternal glucose intolerance that begins after conception, and occurs in the absence of maternal obesity, impairs fertility of male offspring.

Children’s diets are influenced by parental diets, particularly those of the mother^21–23^. Thus, prenatal exposures to poor diet are often compounded by similar postnatal diets. Moreover, many of the effects of developmental exposures are exacerbated by postnatal diet, or only become apparent when the offspring are exposed to additional metabolic challenge^24,25^. Conversely, it has been proposed that developmental exposure to high fat may produce predictive adaptive responses in offspring, such that they may be protected from similar exposures encountered in adulthood^26^. Therefore, the second objective of this study was to determine whether postnatal high fat diet interacts with prenatal exposure to maternal glucose intolerance to affect male fertility. To test this, male offspring were challenged with the same high fat, high sucrose diet that was fed to mothers during pregnancy.

One potential mechanism by which both pre- and postnatal exposure to high fat diet may affect male fertility is germ cell apoptosis. In male rats, there have been conflicting studies on the effects of diet on apoptosis, with postnatal high fat feeding having been shown to both suppress^27^ and increase cell death^28^. Also in the rat, maternal high fat feeding reduces oocyte apoptosis in prepubertal offspring, but ultimately leads to increased apoptosis at 17 weeks of age^25^. Thus, germ cell apoptosis was measured in male offspring shortly after puberty, and at 19 and 31 weeks following exposures to maternal and postnatal high fat diets.

## Results

### 11 Weeks

The effects of *in utero* and post-weaning HFHS diet consumption were first examined in male offspring at 11 weeks of age. There were two maternal diet groups during pregnancy, a standard chow control diet (CD) and a defined high fat, high sucrose diet (HFHS). At weaning at three weeks of age, one male pup from each litter was randomly assigned to the CD, and one to the HFHS diet. The combination of two maternal diet groups and two postnatal pup diet treatment groups resulted in four total groups of offspring, i.e., the CD-CD, CD-HFHS, HFHS-CD, and HFHS-HFHS.

#### Glucose metabolism and body weights in male offspring

Metabolic data for 11 week old male offspring are summarized in Table 1. Postnatal HFHS diet increased fasting glucose concentrations (pup CD group: 102.5 ± 8.3 vs pup HFHS group: 136.0 ± 9.1 mg/dL, P < 0.05), but did not affect the response to a glucose challenge. In pairwise comparisons, fasting blood glucose concentration was lower in the CD-CD group than in the CD-HFHS group, but not in the HFHS-CD group compared to the HFHS-HFHS group (Table 1). In contrast, insulin concentrations were significantly lower in the HFHS-CD group than in the HFHS-HFHS group, but were not different between the CD-CD and the CD-HFHS groups, suggesting that a slight increase in insulin compensated for insulin resistance in the HFHS-HFHS group (Table 1). Post-weaning feeding of HFHS diet to male offspring significantly increased body weights compared to the CD-CD group, with HFHS-CD offspring at an intermediate weight (Table 1).

**Table 1:** Body weight and glucose metabolism parameters in male offspring at 11 weeks of age*.

Treatment	n	Body weight (g)	Fasting glucose (mg/dL)	GTT Area Under Curve	Fasting insulin (ng/ml)
CD-CD	4	24.0 ± 0.8^a^	86.3 ± 12.1^a^	21514.0 ± 1521.1	2.3 ± 0.4^a^
CD-HFHS	4	27.1 ± 0.8^b^	147.8 ± 15.1^b^	24398.8 ± 1317.3	2.0 ± 0.4^ab^
HFHS-CD	7	25.1 ± 0.6^ab^	118.8 ± 9.3^b^	22111.4 ± 995.8	1.3 ± 0.3^b^
HFHS-HFHS	7	26.8 ± 0.5^b^	124.2 ± 8.3^b^	23556.1 ± 995.8	2.7 ± 0.3^a^

*Values are least square means. a,bMeans with different superscripts within a column differ (P < 0.05).

#### Testicular gene expression

**Figure 1:**
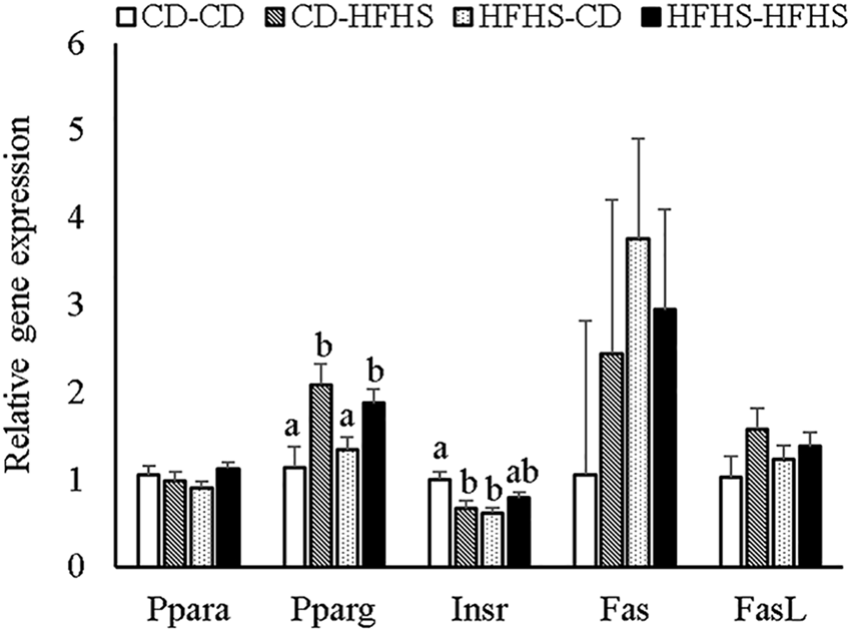
Gene expression of *Ppara*, *Pparg*, *Insr*, *Fas* and *Fasl* in the testis at 11 weeks of age (**a** and **b**: means with different letters were different, P < 0.05).

Real-time semi-quantitative RT-PCR was performed to examine the steady-state mRNA levels in the testis (Fig. 1). Inflammation-associated genes *Tnfa, Il1* and *Il6* were not significantly expressed above negative control levels in any group (data not shown). The transcription factors and master metabolic regulators, peroxisome proliferator-activated receptor -alpha and –gamma were also examined. *Ppara* transcript levels were not affected by either prenatal or postnatal HFHS. *Pparg* also was not affected by maternal diet, but was significantly increased by postnatal HFHS diet feeding (P < 0.01). There was a significant interaction between maternal and postnatal diets in the levels of insulin receptor (*Insr*) mRNA (P < 0.01). Within the maternal CD groups, postnatal HFHS significantly decreased *Insr* levels. Maternal HFHS also decreased *Insr* expression, but postnatal HFHS had no further effect on pups exposed to maternal HFHS. To examine molecular correlates of apoptosis, *Fas* and *Fas* ligand transcript levels were examined, but were not different among the treatment groups. In addition, compared to the CD-CD control, the overall *Insr* level in all HFHS diet feeding groups was lower (P < 0.05).

#### Case of azoospermia in a male exposed to HFHS diet during pregnancy and post-weaning

**Figure 2:**
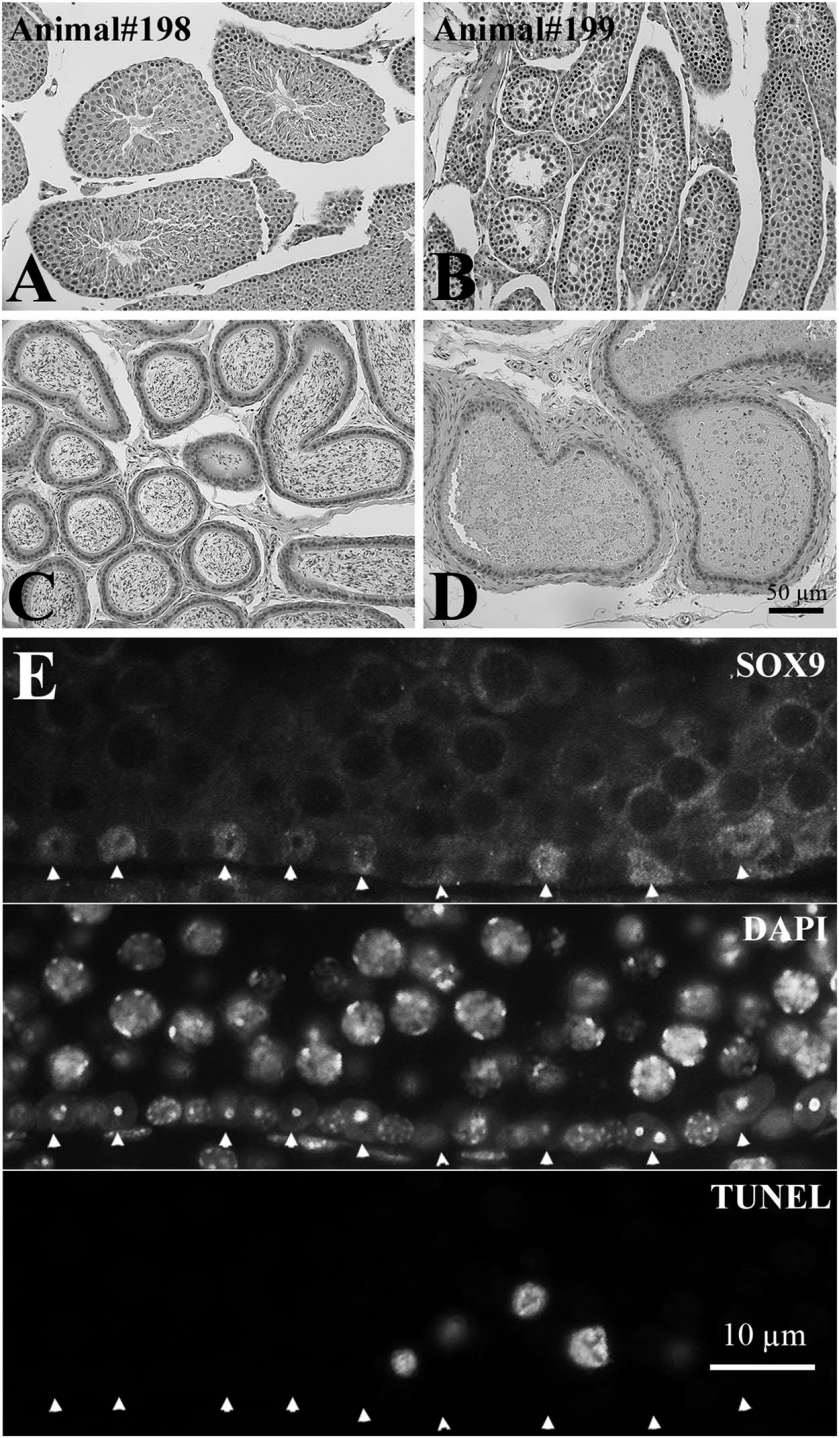
Cross sections of normal (animal #198) and azoospermic (animal #199) testes (**A**,**B**) and epididymides (**C**,**D**). (**E**) Immunostaining of SOX9 (Sertoli cell marker), DNA counterstaining by DAPI, and TUNEL labeling of apoptotic germ cells in the seminiferous tubules of male #199. The spacing of Sertoli cells (arrowheads) along tubule basement membrane appears normal, and the TUNEL positive cells are spermatogonia and spermatocytes, as in other males. Mice #198 and #199 were brothers exposed to HFHS *in utero*. After weaning, mouse 198 was on CD and 199 on HFHS diet.

When testis sections were examined under the microscope for apoptosis analysis (summarized below), it was observed that those from one of the males (animal# 199) from the HFHS-HFHS group did not contain any round or elongating spermatids or spermatozoa (Fig. 2). Spermatocytes were found, and the structure of the seminiferous tubules was normal, although their diameter was reduced (57.7 ± 1.9 µm vs. 100.0 ± 1.8 µm for his sibling, mouse #198). The spacing of Sertoli cells along the tubule basement membrane appeared normal (Fig. 2E). This male’s body weight and serum testosterone (3.3 ng/ml) were normal, falling well within the range of the other animals. The number of TUNEL-positive germ cells per 20 seminiferous tubules was second highest in the HFHS-HFHS group and the TUNEL positive cells (Fig. 2E) were spermatogonia and spermatocytes, like in other males. All other males displayed normal spermatogenesis.

### 19 and 31 Weeks

The four treatment groups were replicated in order to examine sperm counts and testicular morphology in fully mature adult males.

#### At 19 weeks of age

Data on male offspring body weight, testis weight, epididymis weight (whole and cauda only), and sperm numbers recovered from epididymides in the CD-CD, CD-HFHS, and HFHS-CD groups are summarized in Table 2. Body weight in the CD-HFHS group was heavier than the CD-CD and HFHS-CD groups (P < 0.05). No difference was found between the CD-CD and HFHS-CD groups. Testis weight did not differ among the three groups. Cauda and whole epididymis weight in the HFHS-CD group tended to be lower than the CD-CD group (P = 0.1). Adult diet tended to affect the numbers of spermatozoa recovered from epididymides, with some evidence of lower numbers in the CD-HFHS group compared to the CD-CD control (P = 0.08).

**Table 2:** Body, testis, and epididymis weight, and total sperm number recovered from cauda epididymides of the mice from different diet groups at 19 or 31 weeks of age.

Treatment	n	Body wt. (g)	Testis (mg)	Epididymis (mg)	Cauda epididymis (mg)	Sperm count
19w CD-CD	10	29.1 ± 0.6^a^	200.5 ± 4.7	83.0 ± 3.2	30.4 ± 1.5	23.5 × 10^6^ ± 2.1 × 10^6^
19w CD-HFHS	19	32.1 ± 1.1^b^	210.0 ± 3.4	78.8 ± 2.3	28.1 ± 1.1	18.8 × 10^6^ ± 1.6 × 10^6^
19w HFHS-CD	7	28.8 ± 0.8^a^	199.6 ± 5.7	74.1 ± 3.8	26.3 ± 1.8	21.0 × 10^6^ ± 2.6 × 10^6^
31w CD-CD	11	30.1 ± 0.5^a^	195.0 ± 4.0^a^	83.3 ± 3.9^a^	31.9 ± 1.4^a^	39.9 × 106 ± 2.2 × 106^a^
31w CD-HFHS	11	36.0 ± 0.4^b^	200.9 ± 4.1^a,b^	72.2 ± 3.7^b^	27.0 ± 1.3^b,c^	29.0 × 106 ± 2.1 × 106^b^
31w HFHS-CD	7	28.1 ± 0.6^c^	202.3 ± 5.5^a,b^	85.7 ± 5.5^a^	31.6 ± 2.0^a,b^	30.1 × 106 ± 3.1 × 106^b^
31w HFHS-HFHS	5	37.9 ± 0.7^d^	212.3 ± 6.0^b^	77.4 ± 5.5^a,b^	25.4 ± 2.0^c^	21.3 × 106 ± 3.1 × 106^c^

^a,b,c,d^Means with different superscripts, within each age group, within a column differ (P < 0.05).

#### At 31 weeks of age

**Figure 3:**
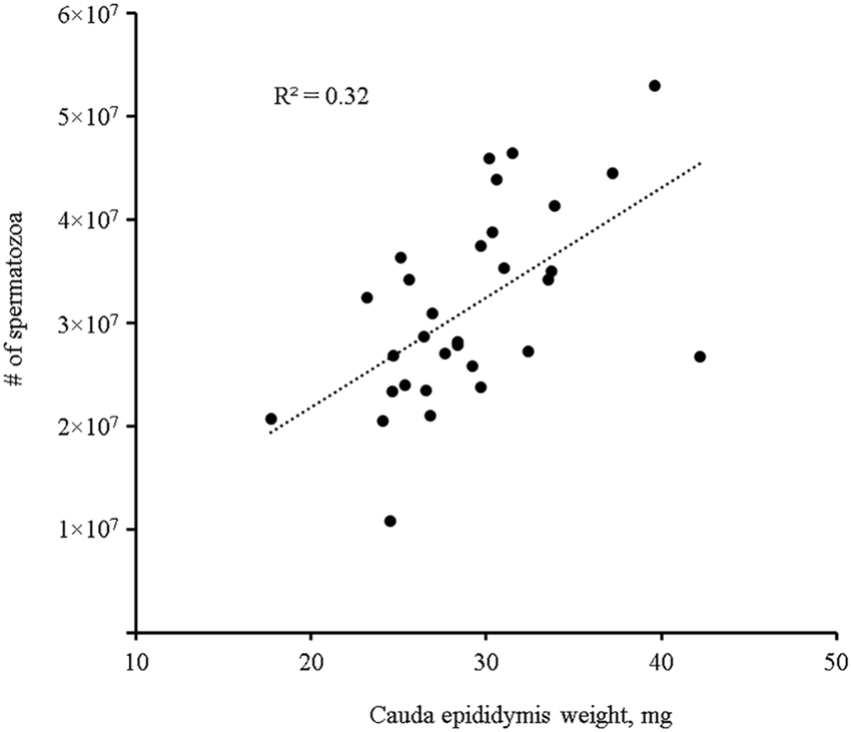
Correlation between the total sperm number recovered from cauda epididymides and the weight of cauda epididymides.

Mouse body, testis and epididymis weights, and total sperm numbers recovered from epididymides at 31 weeks of age are summarized in Table 2. Dam diet alone had no effects on body weights of 31 week old offspring (dam HFHS group: 32.6 ± 0.4 vs CD: 33.2 ± 0.3 g), but offspring diet did (pup HFHS group: 36.6 ± 0.4 vs CD: 29.3 ± 0.4, P < 0.01). Interestingly, there was a significant interaction between dam diet and offspring diet (P < 0.01), resulting in the lowest weights in the HFHS-CD mice, and heaviest in the HFHS-HFHS mice (Table 2). For testis weight, postnatal HFHS diet did not have any effect, while maternal HFHS diet tended to increase testis weight (P = 0.07). For epididymis, maternal diet did not have any effect on weight, but postnatal HFHS diet significantly reduced both whole epididymis (including caput, corpus, and cauda) and cauda epididymis weights (P < 0.05). Both maternal and postnatal HFHS diets significantly reduced the total number of spermatozoa recovered from epididymides (P < 0.01). As shown in Table 2, sperm number was highest in the CD-CD control group, while the HFHS-HFHS group had the lowest sperm number and the CD-HFHS and HFHS-CD groups fell in between them. The total number of spermatozoa recovered from epididymides was correlated with cauda epididymis weight (r = 0.57, r^2^ = 0.32; P < 0.05) as shown in Fig. 3.

### Number of TUNEL-positive testicular germ cells at 11, 19 and 31 weeks of age

**Figure 4:**
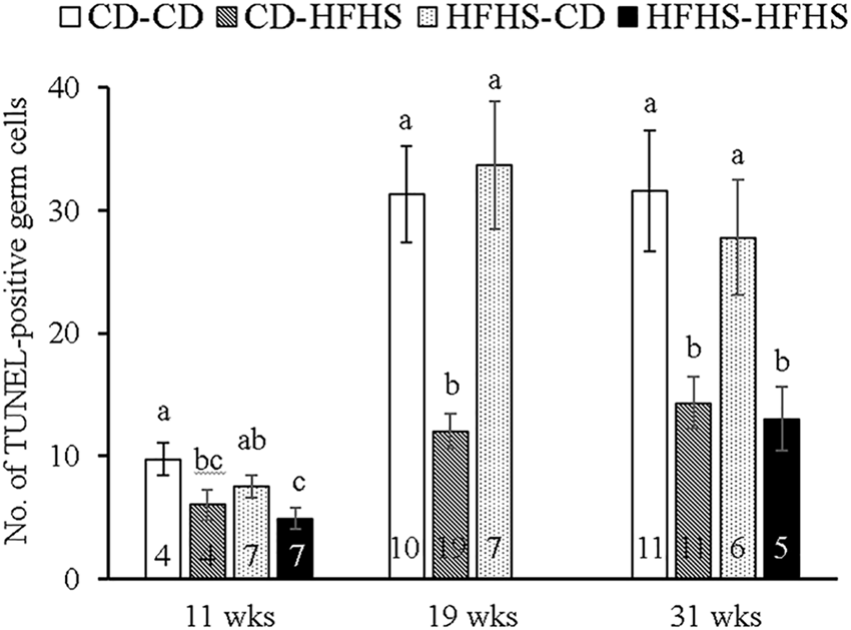
Number of TUNEL-positive germ cells per 20 cross sections of seminiferous tubules in the CD-CD, CD-HFHS, HFHS-CD, and HFHS-HFHS groups at 11, 19, and 31 weeks of age (**a**,**b**, and **c** indicate statistical difference between diets within each age group; means with different letters differ at P < 0.05).

**Figure 5:**
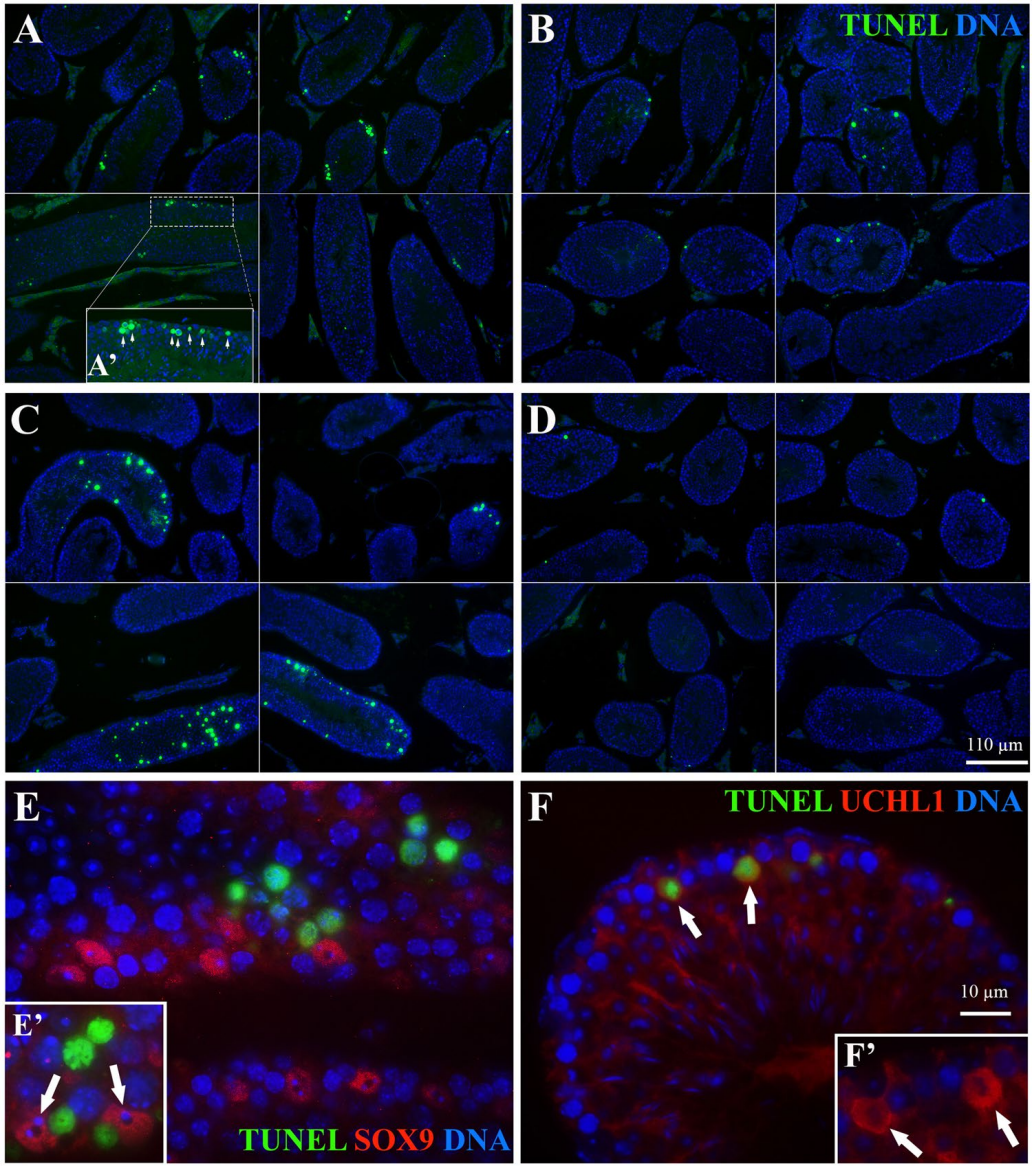
Representative images of TUNEL assay on seminiferous tubule cross-sections from four animals per treatment (**A**: CD-CD, **B**: CD-HFHS, **C**: HFHS-CD, and **D**: HFHS-HFHS diet group). (**E**) Identification of Sertoli cells by anti-SOX9 antibody in red, a Sertoli cell-specific marker (indicated by arrows in E’). SOX9 labeling does not overlap with green fluorescence of TUNEL probe, indicating germ cells, rather than Sertoli cells are apoptotic in the examined tissues. (**F**) Identification of spermatogonia by anti-UCHL1 antibody in red, a specific spermatogonial marker (arrows). Inset F’ shows the blue and red channel detail of two large, green-labeled TUNEL-positive cells visible in panel F. Colocalization of TUNEL labeling and UCHL1 (**F**) indicates that apoptosis occurs in the spermatogonia, close to basement membranes (see also white arrows in panel A’).

Apoptosis of germ cells was evaluated by the TUNEL assay on testis sections from all three ages (Fig. 4). Representative images from each diet group at 11 weeks of age are presented in Fig. 5. The *in utero* HFHS exposure did not affect the number of apoptotic germ cells in any age group, whereas postnatal feeding of HFHS diet for 8 weeks, about two rounds of spermatogenesis^29^, regardless of starting age, significantly reduced the number of apoptotic germ cells (P < 0.05). At 11 weeks, the number of TUNEL-positive germ cells in the CD-HFHS and HFHS-HFHS groups was lower (P < 0.05) than in CD-CD and HFHS-CD groups, respectively. When apoptosis was calculated as a percentage of all spermatogonia, the values of 1.10 ± 0.14, 0.61 ± 0.06, 0.92 ± 0.08, and 0.53 ± 0.05% for the CD-CD, CD-HFHS, HFHS-CD, and HFHS-HFHS groups, respectively, yielded the same statistical results as when calculating the number of TUNEL-positive germ cells per 20 seminiferous tubules. At 19 weeks, the number of TUNEL-positive germ cells in the CD-HFHS group was again lower (P < 0.05) than those in the CD-CD control and HFHS-CD groups. Similarly, at 31 weeks of age, it was lower in the CD-HFHS and HFHS-HFHS groups than in the CD-CD and HFHS-CD groups (P < 0.05); but there were no differences based on *in utero* diets.

Age had a significant effect on the number of TUNEL-positive germ cells (P < 0.01). More specifically, this number was higher at 19 (21.9 ± 2.4) and 31 (22.0 ± 2.4) weeks of age compared to the 11 week age group (6.7 ± 1.5). Co-staining for the Sertoli cell marker SOX9 and spermatogonial marker UCHL1 showed that apoptosis occurred largely in spermatogonia, and a few spermatocytes, but not in Sertoli cells (Fig. 5E,F).

### Serum testosterone concentrations of 11, 19, and 31 week old animals

Serum testosterone (T) concentrations were not different among age groups (Table 3), though there was an interaction between maternal diet and age (P < 0.05). The T concentrations in the older age groups (19 and 31 weeks) were less variable while they were highly variable in the 11 week age group. There were no maternal diet effects, postnatal diet effects or interactions between them in the 19 and 31 week old animals. However, in the 11 week age group, a significant effect of maternal diet on T levels was detected, with maternal HFHS diet reducing T levels in male offspring (the CD maternal offspring: 3.63 ± 1.09 vs. the HFHS maternal offspring: 0.95 ± 0.82 ng/ml, P < 0.05). No correlation was found between serum T concentrations, and body weight, testicular weight or sperm count (not shown).

**Table 3:** Serum testosterone concentrations (ng/ml) in male offspring at 11, 19, and 31 week old.

Treatment	11 weeks	19 weeks	31 weeks
CD-CD	1.87 ± 1.61	1.63 ± 0.08	1.78 ± 0.10
CD-HFHS	5.39 ± 3.13		1.90 ± 0.14
HFHS-CD	0.37 ± 0.06	1.70 ± 0.24	1.83 ± 0.05
HFHS-HFHS	1.52 ± 0.72		1.81 ± 0.06

## Discussion

The main goal of the present study was to test the hypothesis that *in utero* exposure to HFHS-induced maternal glucose intolerance, postnatal HFHS diet and the interaction between them alter spermatogenesis and reduce sperm output in male offspring. We found that feeding of HFHS diet for 8 weeks after weaning suppressed germ cell apoptosis in testes in all age groups studied. While 8 weeks of postnatal feeding of HFHS diet alone significantly increased body weight at all three age points, it had a greater effect on the offspring of glucose intolerant dams than on the offspring of dams fed control diet at 31 weeks of age. High fat diet feeding from 23–31 weeks of age significantly reduced epididymis weight and sperm count, with the lowest count in the HFHS-HFHS group, intermediate counts in the CD-HFHS and HFHS-CD groups, and the highest sperm count in the CD-CD controls. Taken together, these data support the hypothesis that postnatal feeding of high fat diet alters spermatogenesis and reduces sperm production; exposure to glucose intolerance *in utero* exacerbates the effects of an adult high fat diet on weight gain and male fertility.

*In utero* exposure to the HFHS diet reduced the sperm count in 31 week old animals, and seemed to have slightly reduced apoptosis in the 11 week old animals. The combination of postnatal and *in utero* high fat feeding had the most dramatic effects on male offspring fertility. At 31 weeks, HFHS-HFHS had the lowest sperm counts, and testicular and cauda epididymal weights. Although it was an isolated observation, one of seven mice in the HFHS-HFHS exposure group was azoospermic at 11 weeks of age. These data indicate that *in utero* exposure to HFHS diet has negative effects on male offspring fertility, which to our knowledge has not been reported previously and is consistent with prior observations of sperm epigenetic alterations in offspring of obese diabetic mothers^18^.

The cause for the single case of azoospermia at 11 weeks is not clear. Determining whether this was a true effect of the HFHS-HFHS treatment or a chance observation would require a much larger sample size. Body weight, the number of TUNEL-positive germ cells, and the spacing of Sertoli cells along tubule basement membrane in the azoospermic animal were normal and close to those of other animals. Thus, none of these endpoints explain the observed azoospermia.

Direct exposure to HFHS diet after weaning alone affected the weight of epididymides, sperm count and body weight. This is consistent with observations in both human and animal studies. In humans, overweight or obese men with female partners of normal body mass index have increased time to pregnancy interval^8^, and are more likely to suffer from male infertility^4,5^. Furthermore, HFHS-induced paternal obesity impairs embryo development, and decreases pregnancy rates and live birth outcomes^4,30^. In rodents, paternal obesity affects gene expression in male placenta and global DNA methylation status in female placenta^11^,and reduces the fertility of his offspring^13^. Similarly, embryos produced by males fed an HFHS diet have reduced cell numbers^9,10^ and altered metabolism^9^.

These data inspired us to study blood testosterone (T) concentrations as a possible mediator of diet effects on male reproductive system. Overall the testosterone concentrations in the current study were similar to those of the same mouse strain, C57BL/6 J reported by others^31^. T was not abnormally low in the single azospermic male. Though testosterone is so pulsatile^32^ that it is seldom possible to see differences between treatment groups from a single blood sample, we were able to detect significantly lower T in the 11 week old offspring of HFHS-fed dams. There was some indication, though not significant due to the high inter-individual variation, of higher T in the 11 week old males with postnatal HFHS treatment. T concentrations in 19 and 31 weeks old animals were less variable than those in 11 week old animals, and did not differ among diet groups. Thus, the peripubertal period may be especially critical for T in the offspring exposed to glucose intolerance *in utero*, but differences in T do not explain the effects of postnatal diet, or the low sperm counts in the mature offspring of HFHS-fed dams.

Apoptosis is a genetically-controlled, programmed form of cell death required for normal spermatogenesis^33^. During spermatogenesis, apoptosis maintains the size of the germ cell population, optimizes germ cell–Sertoli cell ratio^34^, and eliminates defective germ cells that carry damaged DNA, thus playing a key role in maintaining normal sperm output^35^. In agreement with previous reports^36,37^, apoptosis was present in all animals in this study, and levels were lower in 11 week old group compared to 19 and 31 weeks of age, consistent with previous observations that apoptosis levels increase from early puberty through 4 months of age and then plateau^38^. Most of the apoptotic germ cells were UCHL1^+^ spermatogonia close to the basement membrane. Apoptosis in spermatogonia at early stages of differentiation, specifically at A2, A3 and A4 generations of spermatogonia^37^, is thought to result in early elimination of defective germ cells^39^.

A small percentage of germ cells destined to be eliminated in the testis escape apoptosis and find their way into the ejaculate, a phenomenon called abortive apoptosis^40^. In the current study, we found that postnatal HFHS diet significantly suppressed apoptosis during spermatogenesis. It is possible that this increases the number of defective spermatozoa in ejaculate, but ejaculate was not tested here. A recent study likewise reported suppression of germ cell apoptosis in immature rats fed a high fat diet^27^, whereas others have found increased apoptosis in mature males fed high fat diet^28^. How HFHS diet might inhibit apoptosis in testis is not known. One possible mechanism is via peroxisome proliferator-activated receptor gamma (PPARG). We found that *Pparg* mRNA was upregulated by postnatal HFHS feeding, consistent with a previous study of a 60% fat diet^41^. *Pparg* is a key regulator of metabolism, and its inhibitors can both block and promote apoptosis, depending on cell type^42^. However, *Pparg* function in testicular apoptosis has not yet been studied. Additionally, Sertoli cells tightly regulate germ cell proliferation and differentiation, and control germ cell apoptosis by a FAS/FASL-mediated pathway^43^. Thus, we examined *Fas* and *Fasl* mRNA in the testes in the 11 week old males, but they were not significantly changed by either prenatal or postnatal HFHS.

The reduction in spermatagonial cell death by postnatal HFHS does not explain lower sperm counts in the HFHS-fed males, and while maternal HFHS consumption also decreased offspring sperm counts, it did not affect apoptosis rates. Thus, we examined insulin receptor expression as a potential mechanistic link between HFHS diet and sperm counts. Previous studies have found that insulin and insulin-related proteins are required for FSH-mediated immature Sertoli cell proliferation during the late fetal and early neonatal testicular period^44^. Insulin plays an important role in regulating the final number of Sertoli cells, testis size, and daily sperm output through both systemic and local actions^45,46^. Expression of the insulin receptor was reduced by HFHS diet, whether maternal or postnatal (all HFHS diet groups vs. control) and all HFHS diet groups had reduced sperm count. Therefore, reduced local sensitivity to insulin in the HFHS diet feeding animals may be one of the mechanisms for low sperm output.

In summary, the present study demonstrated that *in utero* high fat diet exposure, consumption of this diet after weaning, and the combination of the two had significant effects on body weight/obesity, reproductive organ weight, and sperm count in the male offspring. These data are consistent with the DOHaD concept that developmental exposures influence male reproductive health.

## Materials and Methods

### Mice

C57BL/6J mice were used for all experiments. Dams were purchased from Jackson Laboratories (Bar Harbor, Maine). Animal experiments were performed at the School of Medicine (experiment 1) and the Animal Sciences Research Center (experiment 2) of the University of Missouri, in compliance with NIH Guidelines for the Care and Use of Laboratory Animals, under protocols approved by the University of Missouri Animal Care and Use Committee.

### Diets

The control diet (CD) was a 5008 chow purchased from LabDiets Purina Mills (PMI Nutrition International LLC, Bentwood, MO). The defined high fat, high sucrose diet (HFHS, D12451) was from Research Diets Inc. (New Brunswick, NJ). The caloric and relative fat contents of these diets are summarized in Table 4.

**Table 4:** Relative energy content of major nutrients in the experimental diets.

	Proportion of calories (%)				
	Fat	Carbohydrate	Sucrose	Protein	Energy density (kCal/g)
5008 Chow	17	66.5	2.4	26.5	4.36
DIO 45 kCal% fat	45	35	17	20	4.73

### Experimental Design

Seven-week old females were assigned randomly to one of the two diet groups: standard control diet or the HFHS diet and mated to proven breeder males after 1 week of acclimation as previously described^20^. The day of copulatory plug visualization was noted as day 0.5 of gestation. After delivery, all dams were placed on the standard chow diet and maintained on this diet until weaning when pups reached 3 weeks old. All pups were sexed at weaning. Only males were kept for the experiments and fed either CD or HFHS diet depending on the experiment design. In experiment 1, at weaning, one male pup from each litter was randomly assigned to the control diet, and one to the HFHS diet. At 11 weeks of age, after a total of 8 weeks on the postnatal CD or HFHS diet, intraperitoneal glucose tolerance tests (GTT) were performed as previously described^20^ with duplicate measurements made using two ReliOn Prime Blood Glucose Monitoring System meters (Walmart, Bentonville, AR). Pups were then sacrificed for collection of testes and epididymides. Tissues were processed as described below. In experiment 2, fifteen dams each were randomly assigned to the CD and HFHS groups, with 12 CD and 6 HFHS dams delivering pups, following a four day breeding window. One male offspring from each litter was assigned to HFHS diet beginning at 23 weeks of age, and one male offspring continued on CD diet, until both were sacrificed at 31 weeks of age with 8 weeks on experimental diet as in experiment 1. Remaining male offspring from each litter were assigned to either continued CD or HFHS beginning at 11 weeks of age, and sacrificed at 19 weeks of age, again 8 total weeks on experimental diet.

### Blood, Tissue and Sperm Collection

At the end of experiments, mice were euthanized by CO_2_ followed by cervical dislocation. Blood was collected by cardiac puncture in a syringe with a 27G needle, stored at 4°C and allowed to clot until serum separation the following day. For experiment 1, testes and epididymides were dissected, immediately placed on wet ice, weighed and fixed for TUNEL staining as described below. For experiment 2, testes and epididymides were collected and weighed. Testes were fixed for apoptosis assay by TUNEL. To recover the cauda-epididymal spermatozoa, epididymides from both sides were placed in 3 ml of warm TCM 199 medium (Corning Life Sciences, Tewksbury, MA), cut into small pieces and incubated at 37°C in a CO_2_ incubator for two periods of 30 min with fresh medium each time, to allow spermatozoa to swim out. The numbers of spermatozoa recovered were then counted by using a hemocytometer.

### Histological and Apoptosis Assessment of Testis Sections by TUNEL

Testes were embedded in paraffin wax after fixation with 4% paraformaldehyde in PBS overnight at 4°C. Serial sections were cut at a thickness of 5 µm, and stained with hematoxylin and eosin at the pathology laboratory of IDEXX BioResearch, Columbia MO. Presumed apoptotic cells in seminiferous tubules on testis cross sections were identified by terminal deoxynucleotidyl transferase (TdT)-mediated nick end labeling (TUNEL) using the *In Situ* Cell Death Detection Kit, AP (Roche, Indianapolis, IN) according to the manufacturer’s instructions. After deparaffinization in xylene, tissue sections were rehydrated in a series of ethanol (from 100% to 50% in water), incubated with proteinase K (20 mg/ml), and permeabilized with freshly prepared 0.1% Triton X-100 in 0.1% sodium citrate. Immediately before use, the TUNEL reaction mixture was prepared by mixing 5 µl of enzyme solution with 45 µl of label solution. Fifty µl of reaction mixture was added on to section and incubated for 1 h at 37°C in a humidified atmosphere in the dark. Negative controls were obtained by the replacement of enzyme solution with PBS. For immunostaining of SOX9 and UCHL1 proteins, after rehydration in series of ethanol, testis sections were permeabilized in PBS containing 0.1% (v/v) Triton X-100, and blocked for 25 min in 0.1 M PBS containing 5% normal goat serum and 0.1% Triton X-100. Samples were incubated overnight at 4°C with primary antibody diluted at 1:200 in 0.1 M PBS containing 1% normal goat serum and 0.1% Triton X-100. On the next day, after a wash in PBS, the primary antibodies (anti-SOX9, catalog# ab185966, purchased from Abcam, Cambridge, MA, and anti-UCHL1 antibodies, catalog# ADI-905-520-1 from Enzo Life Sciences, Ann Arbor, MI) were detected by a mixture of goat anti-rabbit IgG-TRITC diluted at 1:100 and DAPI (4,6-diamidino-2-phenylindole, 2.5 μg/mL) incubated at room temperature for 40 min. Negative controls were obtained by the replacement of primary antibody with normal rabbit serum at the concentration identical to that of specific antibody. At the end of incubation, sections were washed 3 times with PBS and mounted with anti-fade mounting medium (Vector Laboratories Inc., Burlingame, CA). Tissue sections were examined for fluorescence labeling and the acquisition of multiple images of each cross section was performed under a Nikon Eclipse 800 epifluorescence microscope (Nikon Instruments, Melville, NY) with Cool Snap camera (Roper Scientific, Tucson, AZ, USA) and MetaMorph software (v7.1, Universal Imaging, Downington, PA). Images were edited with Adobe Photoshop CS5 (Adobe Systems, Mountain View, CA). Both total and TUNEL-positive spermatogonia were recorded blind to treatment groups. The number of TUNEL-positive germ cells per 20 seminiferous tubules and percentage of positive cells out of total spermatogonia number within tubule section was calculated and used for data analysis.

### RNA isolation and quantitative RT-PCR

RNA was isolated from offspring testes samples collected at 11 weeks of age by homogenization in TRI Reagent (Sigma-Aldrich, St Louis, MO), using a General Laboratory homogenizer (OMNI International, Kennesaw, GA). RNA was purified using the Nucleospin RNA clean-up kit (Clontech Mancherey-Nagel, Mountain View, CA) according to the manufacturer’s protocol. Genomic DNA was eliminated from RNA samples using the Turbo DNA-free kit (ThermoFisher Scientific, Waltham, MA).

Reverse transcription was performed using the SuperScript First-Strand Synthesis System (ThermoFisher Scientific) according to the manufacturer’s protocol. Briefly, 1 µg of RNA was reverse transcribed using random hexamer primers. Relative mRNA levels were quantified by real-time PCR with SYBR Green Master Mix (Qiagen, Germantown, MD) for the following genes: *Pparg, Ppara Insr, FasL* and *Fas*. *Gapdh* and *Actb* were used as the internal reference genes for normalization of gene expression level. PCR was performed on a CFX Connect Real-Time PCR Detection System (Bio-Rad Laboratories, Hercules, CA) with the following cycling conditions: 95°C for 10 minutes (1 rep), and followed by 40 cycles of 95°C (15 s), 60°C (60 s) and 72°C (30 s). Melting curves were generated following RT-PCR to assess the specificity of amplicons. Each sample had only one peak. Thus, no samples were excluded for final analysis.

*Insr* primer sequences have previously been published^47^, and *Actb, Pparg, Ppara, Fas and FasL* primers were designed and synthesized by Integrated DNA Technologies (https://www.idtdna.com/scitools/Applications/RealTimePCR). Primer sequences for the genes were: *Actb*, forward (5′-GATGACCCAGATCATGTTTGAGACC-3′) and reverse (5′-AGATGGGCACAGTGTGGGTGA-3′); *Insr*, forward (5′-CCACCAAGAACTCGTGAAAGG-3′) and reverse (5′-TGCACGCAGGAAAGAACCT-3′); *Gapdh*, forward (5′-TGCACCACCAACTGCTTAGC-3′) and reverse (5′-GGCATGGACTGTGGTCATGAG-3′); *Ppara*, forward (5′-TGCAACTTCTCAATGTAGCCT-3′) and reverse (5′-AATGCCTTAGAACTGGATGACA-3′); *Pparg*, forward (5′-TGCAGGTTCTACTTTGATCGC-3′) and reverse (5′-CTGCTCCACACTATGAAGACAT-3′); *Fas* forward (5′-GTTTGTATTGCTGGTTGCTGT-3′) and reverse (5′-ACCAGACTTCTACTGCGATTC-3′); *Fasl*, forward (5′-ATATGTGTCTTCCCATTCCAGAG-3′) and reverse (5′-CACCAACCAAAGCCTTAAAGTATC-3′). Primer efficiencies were validated by using serial dilutions of the respective template. Reference genes did not differ in expression across the treatment groups. PCR was analyzed using the ΔΔcycle threshold method as described previously^48^, but with adjustment for PCR reaction efficiencies, calculated from dilution series for each gene of interest.

### Serum Analyses

All animals were selected for testosterone (T) assays except the 19 weeks age group, in which only animals from CD-CD and HFHS-CD groups were available for reproductive organ collection at this age. Serum T levels were determined by using T ELISA kit (Immuno-Biological Laboratories Inc., Minneapolis, MN) according to manufacturer’s instructions. Each blood sample was assayed in duplicate. The intra assay coefficients of variation (CV) was 6.4% and inter assay CV was 8.3%.

Intraperitoneal glucose tolerance tests (GTT) were performed as previously described in 11 week old mice of experiment 1^20^. Fasting serum insulin concentrations were measured just prior to glucose tolerance testing by using a Rat/Mouse Insulin ELISA kit (EMD Millipore) in duplicate according to the manufacturer’s instructions. All samples were assessed in one assay. The intra assay CV was 2.6%.

### Statistical Procedures

Mouse glucose tolerance data were analyzed as repeated measures, reading at time 0, 15, 30, 60, and 90 minutes after glucose challenge. The ANOVA model contained the effects of diet, time (minutes after glucose challenge), and the interaction of diet with time (diet × time). The mouse served as the experimental unit. These data were analyzed by using the PROC MIXED procedure in SAS (v 9.4)^49^. Differences in blood glucose concentrations between diet groups over time were determined by the Fisher least-significant difference test.

Data for the body, testis, and epididymis weights, sperm count, number of apoptotic germ cells on testis sections, and serum insulin concentrations were analyzed for normality using the Wilk-Shapiro test^49^. The sperm count data were logarithmically transformed to approach a normal distribution. All dependent variables were analyzed using the general linear model (GLM) procedure of the SAS software, with maternal and pup diet as the main effect and paired dependent sample t-test as options for comparison of brothers on different diet^49^. Mouse body weight data at the end of each experiment were analyzed by using GLM procedure of SAS with the starting body weight at weaning as a covariate. Differences in testis weight, epididymis weight, sperm count, and number of apoptotic germ cells among diet groups were determined by the Fisher least-significant difference. Serum testosterone data and the numbers of TUNEL-positive germ cells per 20 seminiferous tubules from all three age groups were analyzed by using the PROC MIXED procedure in SAS (v 9.4)^49^. The ANOVA model contained the effects of pup age, maternal diet, offspring diet and interaction between them. Offspring were nested within dam, and maternal and offspring diet interactions were used as error term. Gene expression data were analyzed by nested, two-way ANOVA with maternal treatment and offspring diet as the fixed effects and with dam considered a random effect. Linear regression analyses^49^ were also performed to determine the associations between epididymis weight and sperm count. All data are expressed as least squares means ± the standard error of the least squares means.
